# Preliminary Studies on Liquiritin, Deoxyschizandrin, and Tanshinone II A as Potential Anti-Neurodegenerative Disease Agent: Determination by Reverse-Phase Liquid Chromatography in Tianwang Buxin Pills

**DOI:** 10.1155/2019/3150942

**Published:** 2019-08-05

**Authors:** Dan Xu, Sicen Wang, Xiaofang Hou, Changshan Sun

**Affiliations:** ^1^School of Traditional Chinese Medicine, Shenyang Pharmaceutical University, Shenyang 110016, China; ^2^School of Pharmacy, Xi'an Jiaotong University, Xi'an 710061, China; ^3^School of Pharmacy, Shenyang Pharmaceutical University, Shenyang 110016, China

## Abstract

*Tianwang Buxin pill* (TWBXP) is an ancient Chinese classic prescription. Liquiritin, deoxyschizandrin, and tanshinone II A are three bioactive components in TWBXP, which have been proven to be closely related to the therapy effect of neurodegenerative disease. Their contents are very low in TWBXP. In this study, we used a diode array detector (DAD) to perform a full wavelength scanning in order to choose a most suitable detection wavelength to establish an HPLC method for the simultaneous determination of these three components in TWBXP. Various chromatographic conditions were investigated to verify its applicability. Finally, a Kromasil C18 column (250 × 4.6 mm, 5 *μ*m) thermostated at 30°C, mobile phase as 0.2% phosphoric acid solution (eluent A), and 0.1% phosphoric acid-acetonitrile solution (eluent B) were used. Both external standard method and internal standard method were used for quantification. The results showed that both methods were simple and convenient in operation without special pretreatment and exhibits excellent precision, repeatability (RSD < 3.0%), good linearity (*R*^2^ > 0.9990), and good recoveries (recovery value between 95% and 105%). Because of the low contents in samples, the internal standard method provided a better accurate result than the external standard method. The stability results showed the sample became stable within 24 hours at room temperature. The method provides a convenient and effective way for the quality control of TWBXP, and it can help the research about AD in the future.

## 1. Introduction

*Tianwang Buxin pill* (TWBXP) is an ancient Chinese classic prescription, which was recorded in *She Sheng Mi Pou* of Ming Dynasty. The prescription has been passed down to the present day with exquisite and excellent curative effect on treating heart-yin deficiency, heart palpitations, forgetfulness, insomnia, much dream, dry stool, etc [1]. With the increasing attention and research on TWBXP, many researchers have discovered that TWBXP possessed various significant effects such as antioxidant [2], antiaging [3, 4], antidepressant [5], and cardiovascular protecting effects [6–8]. Therefore, TWBXP has a huge market in Chinese. According to the record, it is composed of 16 traditional Chinese medicines (TCMs).

Three bioactive components in TWBXP, liquiritin, deoxyschizandrin, and tanshinone II A, have been proven to be closely related to the therapy effect of neurodegenerative disease. Liquiritin is a major ingredient in *Glycyrrhizae radix* which has been proven to significantly ameliorate spatial learning, cognitive ability, and memory impairment of rats with Alzheimer's disease [9]. It possessed various pharmacological activities and exhibited various positive biological effects, including neuroprotective [10], anticancer [11], and antidepressant [12] effect. Deoxyschizandrin is a lignans ingredient in *Schisandrae Chinensis Fructus*, with antiaging effect [13], hepatoprotective activity [14] and anticancer effect [15], and it has been experimentally proven that it can significantly improve A*β*_1–42_-induced short-term and spatial memory impairments in the Y-maze and water maze tests [16]. Tanshinone II A is a major ingredient in *Salviae Miltiorrhizae Radix et Rhizoma* which has strong antioxidative activity [17] and neuroprotective effect [18–20]. It can improve the cognitive dysfunction in learning and memory exhibited and ameliorating spatial memory impairment by the AD rats. The possible mechanism is tanshinone II A could ameliorate the synaptic deficit at the early phase of AD and attenuates AD-related protein expression [21–25].

Due to the pharmacological effects of liquiritin, deoxyschizandrin, and tanshinone II A, the simultaneous determination of three bioactive components in TWBXP is significant to disclose the secret underlying its efficacy on neurodegenerative disease. Sun et al. determined 6 lignans (schisandrin, schisandrol B, schisantherin A, deoxyschisandrin, schisandrin B, and schisandrin C) in TWBXP [26] by RPLC. Yan et al. determined four components (tanshinone II A, cryptotanshinone, salvianolic acid B, and tanshinone I) of TWBXP by UPLC-Q-TOF-MS [27]. So far, as we know, no one has reported about simultaneous determination of the components related to AD treatment.

In this study, we established an HPLC method for the simultaneous determination of these three components in TWBXP. Various chromatographic conditions were investigated to verify its applicability. According to the previous experiments, the three active component contents were very low. Quantification of these three components was verified with both the internal standard method and external standard method. The method provides a convenient and effective way for the quality control of TWBXP, and it can help the research about AD in the future.

## 2. Experiments

### 2.1. Instruments

1100 model Agilent HPLC was equipped with LC-G13 solvent delivery unit, G1313A autosampler, G1315B diode array detector, and LC chromatography workstation. Kromasil C18 column (250 × 4.6 mm, 5 *μ*m) and Agilent C18 column (4.6 × 250 mm, 5 *μ*m) were used. BT25S model electronic scale was from Sartorius. KQ5200E model ultrasonic cleaner was from Kunshan Ultrasonic Instrument Co., Ltd. HH-2 model digital circulating water bath was from Jintan Ronghua Instrument Manufacturing Co., Ltd. RE52CS model rotary evaporator was purchased from Shanghai Yarong Biochemical Instrument Factory. ZDHW model heating mantle and 101 model electric blast-drying oven were from Beijing Zhongxing Weiye Instrument Co., Ltd. High speed crusher was from Tianjin Taisite Instrument Co., Ltd.

### 2.2. Materials

HPLC-grade acetonitrile was supplied by Sigma-Aldrich (USA). Analytical grade ethanol was supplied by the Yuwang Group (Shandong). Phosphoric acid was Tianjin Hengxing Chemical Preparation Co. Ltd. Liquiritin (purity >98%), deoxyschizandrin (purity >98%), tanshinone II A (purity >98%), and berberine (purity >98%) reference substances were purchased from China National Institute for the Control of Pharmaceutical and Biological Products. Tianwang Buxin pills were purchased from Beijing Tongrentang Group.

### 2.3. Sample Treatment

The stock solutions of liquiritin (500 *μ*g/mL), deoxyschizandrin (300 *μ*g/mL), tanshinone II A (500 *μ*g/mL), and the internal standard (IS) berberine (1500 *μ*g/mL) were prepared by dissolving suitable quantities of the standard substance in methanol. The series mixed standard working solutions for the standard curve were prepared by diluting the stock solution of liquiritin, deoxyschizandrin, and tanshinone II A at six concentration levels corresponding to 10, 20, 50, 75, 100, and 150 *μ*g/mL of liquiritin; 15, 30, 75, 112.5, 150, and 180 *μ*g/mL of deoxyschizandrin; and 5, 25, 55, 90, 125, and 150 *μ*g/mL of tanshinone II A. IS was spiked into the mixed standard working solutions at 750 *μ*g/mL. All the standard working solutions were labeled as 1, 2, 3, 4, 5, and 6 and stored at 4°C before use.

TWBXP was cut into little pieces, accurately weighted (about 9 g) and transferred into the 100 mL round-bottom flask. They were extracted with 50 mL methanol in an ultrasonic bath for 30 min and diluted to 50 mL by methanol. All samples were filtered through a 0.22 *μ*m microporous membrane syringe filter (Shenyang Chromatography Scientific Instrument Co., Ltd.) and spiked with 750 *μ*g/mL berberine before injecting into the HPLC system for analysis.

### 2.4. Establishment of an HPLC Method for the Determination of Three Active Components

The analyses were carried out on an 1100 model Agilent HPLC equipped with the LC-G13 solvent delivery unit, G1313A autosampler, G1315B diode array detector, and LC chromatography workstation. After optimization of detection wavelength, column type, and elution gradient (Table 1), the chromatographic separation was performed using a Kromasil C18 column (250 × 4.6 mm, 5 *μ*m) thermostated at 30°C, mobile phase as 0.2% phosphoric acid solution (eluent A), and 0.1% phosphoric acid-acetonitrile solution (eluent B). The gradient elution program was from 0% to 15% B in 10 min, was from 0% to 30% B at 30 min, was from 0% to 75% B at 70 min, remained at 75% B to 85 min, returned to 0% B at 86 min, and was in column equilibration for 14 min. The wavelength was set at 203 nm. The injection volume was 5 *μ*L, and the flow rate was set at 1 mL/min.

### 2.5. Validation of the HPLC Method

#### 2.5.1. External Standard Method

To validate the HPLC method for determination of liquiritin, deoxyschizandrin, and tanshinone II A in TWBXP, the specificity, linearity, precision, accuracy, and repeatability of the method were studied. A volume of 5 *μ*L of each mixed standard working solution (1, 2, 3, 4, 5, and 6) was injected under the operating chromatographic conditions previously described. The calibration curve was established by plotting the peak area versus the corresponding concentration.

#### 2.5.2. Internal Standard Method

5 *μ*L of each series mixed standard working solution (1, 2, 3, 4, 5, and 6) spiked with 750 *μ*g/mL berberine was injected under the operating chromatographic conditions previously described. Linearity of each calibration curve was determined by plotting the peak area ratio of analytes to IS versus the concentration of analytes to IS with weighted linear regression.

Certain acceptance limits were developed for linearity, precision, repeatability, and accuracy. The LODs and LOQs of liquiritin, deoxyschizandrin, and tanshinone II A were estimated based on the signal-to-noise ratio criterion S/N = 3 and 10, respectively. The correlation coefficient for linearity, limits of precision and repeatability, and accuracy value has all been calculated.

The percent recovery value was calculated using the equation below:

(1) $$\begin{array}{c} \text{recovery}=\frac{\left(\text{found amount}-\text{initial amount}\right)}{\text{added amount}}\cdot 100\% \end{array}$$

### 2.6. Stability Study of Tianwang Buxin Pills Preparations

The stability of TWBXP solution stored at room temperature was studied at 0, 2, 4, 8, 12, and 24 h, respectively. The stability limit was set to be <3.0% at RSD (%) in 24 hours at 25°C and 40% RH.

### 2.7. Determination of the Real Samples

Eight batches of the TWBXP sample were prepared according to 2.3. The sample solutions (5 *μ*L) spiked with 750 *μ*g/mL berberine were injected into the HPLC system; the contents of liquiritin, deoxyschizandrin, and tanshinone II A in TWBXP were determined by both external standard and internal standard methods.

## 3. Results and Discussion

### 3.1. Optimization of the Chromatographic Separation

**Figure 1:**
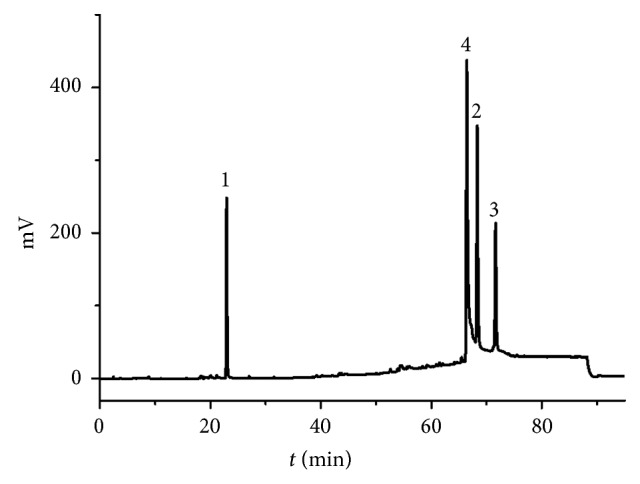
HPLC chromatogram of the mixed standard solution of liquiritin (1), deoxyschizandrin (2), tanshinone II A (3), and internal standard berberine (4).

Columns, UV wavelength, and elution gradient were studied in our work (Table 1). Considering the absorption characteristics of liquiritin, deoxyschizandrin, and tanshinone II A, 203 nm was chosen as the detection wavelength. Optimization of the column type and elution gradient results is shown in Table 1. Using the optimized conditions, linearity, precision, repeatability, and accuracy were tested. The representative chromatograms of the mixed standard are illustrated in Figure 1. All peaks were baseline separated and had a good resolution (*R* ≥ 1.5) under the chromatographic conditions. The retention time of standard liquiritin, deoxyschizandrin, and tanshinone II A was 22.8 min, 68.2 min, and 71.7 min, respectively.

### 3.2. Method Validation

The linearity of the method was evaluated at *λ* = 203 nm using the mixed standard solutions. The results including the regression equations, the linear ranges, and regression coefficients are summarized in Tables 2 and 3.

For linearity, the correlation coefficient (*R*^2^) was above 0.99 by both the external standard method and internal standard method. The precision (Table 4) and repeatability (Table 5) were tested. In the precision experiment, the RSD value of liquiritin, deoxyschizandrin, and tanshinone II A was 1.23%, 1.88%, and 1.43%, respectively (*n* = 6). When it comes to repeatability, the mean concentration of liquiritin, deoxyschizandrin, and tanshinone II A in TWBXP was 0.141 mg/g, 0.129 mg/g, and 0.308 mg/g, while the RSD of which was 1.76%, 2.00%, and 1.95%, respectively.

Recovery studies of liquiritin, deoxyschizandrin, and tanshinone II A were evaluated by analysis of samples of each analyte. The data were calculated with equation (1) and are summarized in Table 6. The recovery range of liquiritin, deoxyschizandrin, and tanshinone II A was 99.22%∼102.92%, 100.48%∼105.28%, and 96.27%∼100.03%, respectively. The RSD value was 1.50%.

### 3.3. Stability Study

The stability test result is shown in Table 7; the RSD value of liquiritin, deoxyschizandrin, and tanshinone II A was 1.18%, 1.34%, and 1.88%, respectively. The results indicate the sample becomes stable within 24 hours at 25°C and 40% RH.

### 3.4. Sample Analysis and Comparison of Two Methods

**Figure 2:**
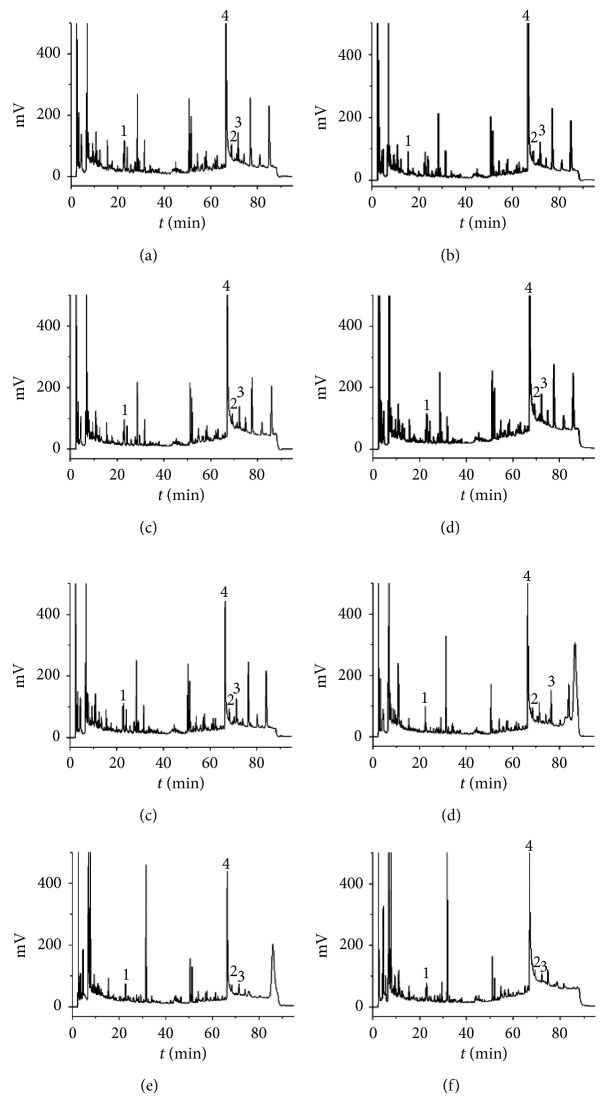
HPLC chromatograms of eight batches of TWBXP samples: (a) 17052802; (b) 17052803; (c) 17070507; (d) 17091903; (e) 17102402; (f) 17015269; (g) 17055293; (h) 18015225. Peak 1, liquiritin; peak 2, deoxyschizandrin; peak 3, tanshinone II A; peak 4, berberine (IS).

The chromatograms of eight batches samples are shown in Figure 2. The peaks of liquiritin, deoxyschizandrin, and tanshinone II A are labeled as 1, 2, and 3.  Table 8 shows the contents of these three active components: liquiritin, deoxyschizandrin, and tanshinone II A under the external standard method and internal standard method, respectively.

Normally, the internal standard method is used in biological samples or some other matrix samples. In this case, we can see that the contents of these three active components are very low in the TWBXP samples. The contents determined by the internal standard method are higher than that by the external standard method. Therefore, it can be concluded that when the contents of analytes are very low, the internal standard method is preferred to be chosen.

## 4. Conclusion

Two methods for the simultaneous determination of the contents of liquiritin, deoxyschizandrin, and tanshinone II A in TWBXP by HPLC was developed and compared in this study. Both of the proposed method offers the following advantages: the method was simple and convenient in operation without special pretreatment and exhibits excellent precision, repeatability (RSD < 3.0%), good linearity (*R*^2^ > 0.9990), and good recoveries (the recovery value between 95% and 105%). However, when the analytes encountered with low content in the real TCM samples, the internal standard method is superior to the external standard method.

## Figures and Tables

**Table 1:** Optimization of gradient conditions.

Chromatographic factor	Conditions	Optimized condition	Reason
Elution gradient	(1) 0–10 min, 0–15% B; 10–30 min, 15–30% B; 30–70 min, 30–75%; 70–85 min, 75% B; 85–86 min, 75–0% B; 86–100 min, 0% B;	Procedure (1)	High peak resolution
	(2) 0–10 min, 0–15% B; 10–30 min, 15–30% B; 30–60 min, 30–65%; 60–70 min, 65–70% B; 70–85 min, 70–75% B; 85–86 min, 75–0% B, 86–100 min, 0% B		

**Table 2:** The Calibration curve of liquiritin, deoxyschizandrin, and tanshinone II A under the external standard method.

	Range (*μ*g/mL)	Slope (mean ± SD)^b^	Intercept (mean ± SD)^b^	*R* ^2^	LOD^a^ (*μ*g/mL)	LOQ^a^ (*μ*g/mL)
Liquiritin	10.00–150.00	7.20 ± 0.41	23.14 ± 1.52	0.9994	3	10
Deoxyschizandrin	15.00–180.00	9.45 ± 0.11	21.77 ± 0.62	0.9984	5	15
Tanshinone II A	5.00–150.00	6.07 ± 0.06	30.90 ± 1.05	0.999	1.5	4.5

^a^Limit of detection (LOD) at S/N = 3 and limit of quantification (LOQ) at S/N = 10. ^b^Data are presented as mean ± SD (*n* = 3).

**Table 3:** The calibration curve of liquiritin, deoxyschizandrin, and tanshinone II A under the internal standard method.

	Range (*μ*g/mL)	Slope (mean ± SD)^a^	Intercept (mean ± SD)^a^	*R* ^2^
Liquiritin	10.00–150.00	(1.5 ± 0.1) × 10^−3^	(4.8 ± 0.3) × 10^−3^	0.9994
Deoxyschizandrin	15.00–180.00	(1.9 ± 0.1) × 10^−3^	(4.8 ± 0.2) × 10^−3^	0.9984
Tanshinone II A	5.00–150.00	(1.2 ± 0.1) × 10^−3^	(6.3 ± 0.2) × 10^−3^	0.9992

^a^Data presented as mean ± SD (*n* = 3).

**Table 4:** The precision test results (*n* = 6).

Number	Liquiritin			Deoxyschizandrin			Tanshinone II A		
	Peak area	Average	RSD (%)	Peak area	Average	RSD (%)	Peak area	Average	RSD (%)
1	300.90	298.57	1.23	659.60	678.33	1.88	270.30	273.37	1.43
2	299.40			684.10			276.90		
3	299.30			684.80			268.60		
4	291.20			664.70			273.80		
5	300.40			688.70			278.80		
6	300.20			688.10			271.80		

**Table 5:** The repeatability test results (*n* = 6).

Number	Concentration (mg/g)		
	Liquiritin	Deoxyschizandrin	Tanshinone II A
1	0.142	0.132	0.309
2	0.145	0.129	0.311
3	0.141	0.126	0.315
4	0.139	0.126	0.310
5	0.14	0.131	0.302
6	0.138	0.127	0.299
Average	0.141	0.129	0.308
RSD (%)	1.76	2.00	1.95

**Table 6:** Recovery results of the three biocomponents (*n* = 6).

Compound	Sample (mg)	Initial amount (*μ*g)	Added amount (*μ*g)	Found amount (*μ*g)	Recovery (%)	Average recovery (%)	RSD (%)
Liquiritin	15.98	11.37	10.00	21.64	102.39	100.83	1.52
	15.95	11.35		21.47	100.93		
	15.92	11.33		21.28	99.22		
	15.71	11.18		21.36	99.93		
	15.65	11.13		20.00	102.92		
	15.42	11.37		21.32	99.58		

Deoxyschizandrin	15.98	6.18	5.00	11.42	105.28	103.81	1.66
	15.95	6.17		11.36	104.08		
	15.92	6.16		11.34	103.68		
	15.71	6.07		11.40	104.88		
	15.65	6.05		11.38	104.48		
	15.42	5.96		11.18	100.48		

Tanshinone II A	15.98	29.05	25.00	53.50	98.05	97.84	1.50
	15.95	28.99		53.08	96.35		
	15.92	28.94		53.06	96.27		
	15.71	28.56		53.36	97.47		
	15.65	28.45		54.00	100.03		
	15.42	28.03		53.71	98.87		

**Table 7:** The stability test results (*n* = 6).

Time	Peak area		
	Liquiritin	Deoxyschizandrin	Tanshinone II A
0 h	438.41	349.33	927.68
2 h	445.9	344.85	933.49
4 h	440.9	345.62	945.12
8 h	438.23	337.05	930.59
12 h	441.56	348.9	907.35
24 h	430.28	341.93	898.63
Mean	439.21	344.61	923.81
RSD (%)	1.18	1.34	1.88

^*∗*^Temperature: 25°C; RH: 40%.

**Table 8:** Contents of liquiritin, deoxyschizandrin, and tanshinone II A by the internal standard and external standard method in eight batches of TWBXP samples (*n* = 6, mean ± S.D.).

Number	Content (mg/g)			
	Analysis method	Liquiritin	Deoxyschizandrin	Tanshinone II A
17052802	External standard	0.16 ± 0.01	0.15 ± 0.01	0.49 ± 0.01
	Internal standard	0.22 ± 0.01	0.28 ± 0.01	0.67 ± 0.01
17053803	External standard	0.14 ± 0.02	0.12 ± 0.02	0.42 ± 0.01
	Internal standard	0.19 ± 0.02	0.22 ± 0.02	0.58 ± 0.01
17070507	External standard	0.15 ± 0.01	0.11 ± 0.03	0.47 ± 0.01
	Internal standard	0.20 ± 0.01	0.19 ± 0.03	0.64 ± 0.01
17091903	External standard	0.17 ± 0.02	0.13 ± 0.02	0.41 ± 0.02
	Internal standard	0.23 ± 0.02	0.23 ± 0.02	0.55 ± 0.02
17102402	External standard	0.16 ± 0.01	0.14 ± 0.01	0.45 ± 0.01
	Internal standard	0.22 ± 0.01	0.21 ± 0.01	0.53 ± 0.01
17015269	External standard	0.15 ± 0.01	0.12 ± 0.02	0.40 ± 0.02
	Internal standard	0.20 ± 0.01	0.20 ± 0.02	0.56 ± 0.02
17055293	External standard	0.13 ± 0.01	0.11 ± 0.03	0.38 ± 0.03
	Internal standard	0.17 ± 0.01	0.25 ± 0.03	0.50 ± 0.03
18015225	External standard	0.11 ± 0.01	0.11 ± 0.03	0.36 ± 0.03
	Internal standard	0.14 ± 0.01	0.19 ± 0.03	0.61 ± 0.03

## Data Availability

The data used to support the findings of this study are included within the article.
